# Supplementary material for: Clinical Characteristics of Adenovirus Pneumonia in Children
Source: Pathogens. 2025 Oct 31;14(11):1110. doi: 10.3390/pathogens14111110 (PMC12655012; doi:10.3390/pathogens14111110)
Supplement: Supplementary file 1 [file pathogens-14-01110-s001.zip › pathogens-3928935-supplementary.pdf]

statistical code

```
##### Analyze the association between age, gender, co-infections and severe disease
# Read CSV file
library(readr)
data <- read.csv("chif test data.csv")

# View the first few rows of the data
head(data)

# View data structure
str(data)
## Convert appropriate columns to factors
data$gender <- as.factor(data$gender)
data$infection <- as.factor(data$infection)
data$group <- as.factor(data$group)
## Statistical Analysis
#a. Difference in group by gender
# Chi-squared test
table_gender_group <- table(data$gender, data$group)
chisq_test_gender_group <- chisq.test(table_gender_group)
print(chisq_test_gender_group)

#b. Difference in group by age
#
table_age_group <- table(data$age, data$group)
chisq_test_age_group <- chisq.test(table_age_group)
print(chisq_test_age_group)


## Analyze differences in group by infection status
# Chi-squared test
table_infection_group <- table(data$infection, data$group)
chisq_test_infection_group <- chisq.test(table_infection_group)
print(chisq_test_infection_group)


# Summarize results into a report
# Aggregate results
results <- list(
  Gender_Group_ChiSq = chisq_test_gender_group,
  Age_Group_ChiSq = chisq_test_age_group,
  Infection_Group_ChiSq = chisq_test_infection_group
)
```

```r
# Print results
print(results)

result_df <- data.frame(
  Test = c("chisq_test_gender_group", "chisq_test_age_group", "chisq_test_infection_group"),
  Statistic = c(chisq_test_gender_group$statistic, chisq_test_age_group$statistic, chisq_test_infection_group$statistic),
  p_value = c(chisq_test_gender_group$p.value, chisq_test_age_group$p.value, chisq_test_infection_group$p.value),
  Degrees_of_Freedom = c(chisq_test_gender_group$parameter, chisq_test_age_group$parameter, chisq_test_infection_group$parameter)
)

# Write results to CSV file
write.csv(result_df, file = "chi_squared_results_combined.csv", row.names = FALSE)

### Calculate Standard Deviation
# Read CSV file
library(readr)
df <- read.csv("E:/ADV/degreeNSAPSD.csv")

# Calculate standard deviation for each subject, ignoring missing values
sd_per_subject <- sapply(df, function(x) sd(x, na.rm = TRUE))

# Print standard deviation for each subject
print(sd_per_subject)



# 1. Read CSV file
df <- read.csv("E:/ADV/degreeSAPSD.csv")

# 2. Calculate standard deviation and mean for each subject
sd_per_subject <- sapply(df, function(x) sd(x, na.rm = TRUE))
mean_per_subject <- sapply(df, function(x) mean(x, na.rm = TRUE))

# 3. Create a data frame to store results
results <- data.frame(
  Subject = colnames(df),
  Standard_Deviation = sd_per_subject,
  Mean = mean_per_subject
)
```

```r
# 4. Write results to a new CSV file
write.csv(results, "E:/ADV/degreeSAPSDdata.csv", row.names = FALSE)



#### Perform Chi-squared test and create grouped comparison plots
# Load necessary packages
library(ggplot2)
library(dplyr)
library(tidyr)
library(broom)

# Read CSV file
df <- read.csv("E:/ADV/chif test data.csv")

# Load necessary libraries
library(ggplot2)
library(dplyr)

# Read data
data <- read.csv("E:/ADV/chif test data.csv")

# Gender Chi-squared test
gender_table <- table(data$gender, data$group)
gender_chisq <- chisq.test(gender_table)

# Age Chi-squared test
age_table <- table(data$age, data$group)
age_chisq <- chisq.test(age_table)

# Infection Chi-squared test
infection_table <- table(data$infection, data$group)
infection_chisq <- chisq.test(infection_table)

# Gender plot
p_gender <- ggplot(data, aes(x = gender, fill = group)) +
  geom_bar(position = "stack", width = 0.7) +
  labs(title = paste("nChi-square p-value:", round(gender_chisq$p.value, 4)),
       x = "Gender",
       y = "Number of Cases",
       fill = "Type") +
  theme_minimal()

# Age plot
```

```
p_age <- ggplot(data, aes(x = age, fill = group)) +
  geom_bar(position = "stack", width = 0.7) +
  labs(title = paste("nChi-square p-value:", round(age_chisq$p.value, 4)),
       x = "Age",
       y = "Number of Cases",
       fill = "Type") +
  theme_minimal()

# Infection plot
p_infection <- ggplot(data, aes(x = infection, fill = group)) +
  geom_bar(position = "stack", width = 0.7) +
  labs(title = sprintf("nChi-square p-value: %.4g", infection_chisq$p.value),
       x = "Infection Type",
       y = "Number of Cases",
       fill = "Type") +
  theme_minimal()

# Display plots
print(p_gender)
print(p_age)
print(p_infection)


########## Perform Wilcoxon rank sum test to calculate P-values etc. and save CSV
##########
library(ggplot2)
library(dplyr)
library(grid)
library(gridExtra)

# Read data
data <- read.csv("Wilcoxon rank sum test data.csv")

# Replace special characters in column names
names(data) <- gsub("—", "-", names(data))  # Replace em dash with en dash or other
appropriate character

# Ensure 'type' column in data is a factor
data$type <- as.factor(data$type)

# Define a function to perform Wilcoxon rank sum test and return detailed results
perform_wilcoxon_test <- function(var, group) {
  test_result <- wilcox.test(data[[var]] ~ data[[group]])
```

```r
  # Calculate median and IQR for each group for reporting
  group_levels <- levels(data[[group]])
  group1_median <- median(data[[var]][data[[group]] == group_levels[1]], na.rm = TRUE)
  group1_iqr <- IQR(data[[var]][data[[group]] == group_levels[1]], na.rm = TRUE)
  group2_median <- median(data[[var]][data[[group]] == group_levels[2]], na.rm = TRUE)
  group2_iqr <- IQR(data[[var]][data[[group]] == group_levels[2]], na.rm = TRUE)

  return(list(
    p_value = test_result$p.value,
    statistic = test_result$statistic,
    group1_median = group1_median,
    group1_iqr = group1_iqr,
    group2_median = group2_median,
    group2_iqr = group2_iqr,
    group1_name = group_levels[1],
    group2_name = group_levels[2]
  ))
}

# Define function to plot Wilcoxon results, adding numbering
plot_wilcoxon_results <- function(data, var, p_value, letter, auc_value) {
  # Create dynamic Y-axis title
  y_label <- switch(var,
                    "PLT" = "PLT (10^9/L)",
                    "CRP" = "CRP (mg/L)",
                    "PA" = "PA (mg/L)",
                    "IFN" = paste("IFN-γ (pg/mL)"), # Dynamically generate Y-axis label
                    "PLR" = "PLR",
                    "CPAR" = "CPAR",
                    "PCT" = "PCT (ng/mL)",
                    "PNR" = "PNR",
                    "NLR" = "NLR",
                    "LYM" = "LYM (10^9/L)",
                    "NEV" = "NEV (10^9/L)",
                    "IL.2" = "IL-2 (pg/mL)",
                    "IL.4" = "IL-4 (pg/mL)",
                    "IL.6" = "IL-6 (pg/mL)",
                    "IL.10" = "IL-10 (pg/mL)",
                    "TNF" = "TNF (pg/mL)",
                    var)   # If var not in switch, use original var

  # Print y_label for debugging
  print(paste("Y-axis label:", y_label))
```

```r
  p <- ggplot(data, aes_string(x = "type", y = var, fill = "type")) +
    geom_boxplot() +
    labs(title = sprintf("p=%.4f", p_value),   # Modify title format to four decimal places
         x = NULL,
         y = y_label) +   # Use dynamically generated y-axis label
    theme_minimal() +
    theme(
      plot.title = element_text(size = 14, hjust = 0.5),      # Modify title font size and
alignment
      axis.title.x = element_text(size = 12), # Modify x-axis title font size
      axis.title.y = element_text(size = 12), # Modify y-axis title font size
      axis.text.x = element_text(size = 12),   # Modify x-axis tick label font size
      axis.text.y = element_text(size = 10),   # Modify y-axis tick label font size
      legend.position = "none",
      panel.grid.major = element_blank(), # Remove major gridlines
      panel.grid.minor = element_blank(),   # Remove minor gridlines
      axis.line = element_line(color = "black"),   # Keep axis lines
      axis.ticks.y = element_line(color = "black", size = 0.5), # Add Y-axis tick marks
      plot.margin = margin(5, 5, 5, 5, "mm")   # Adjust plot margins
    ) +
    # Place letter identifier on the left side of Y-axis
    annotate("text", x = 0, y = Inf, label = letter,
             size = 5, fontface = "bold", color = "black",
             hjust = 1, vjust = -0.5) +   # Adjust hjust and vjust
    coord_cartesian(clip = 'off') # Ensure text is not clipped
  return(p)
}

# Create data frame to store Wilcoxon test results
wilcoxon_results <- data.frame(
  Variable = character(),
  W_Statistic = numeric(),
  P_Value = numeric(),
  Group1_Median = numeric(),
  Group1_IQR = numeric(),
  Group2_Median = numeric(),
  Group2_IQR = numeric(),
  Group1_Name = character(),
  Group2_Name = character(),
  stringsAsFactors = FALSE
)

# Iterate over all variables and perform Wilcoxon rank sum test and plotting
plot_list <- list()
```

```
letters <- LETTERS[1:16]   # From A to P

# Ensure all data column names exclude 'type'
variable_names <- names(data)[!names(data) %in% "type"]

for (i in seq_along(variable_names)) {
  var <- variable_names[i]
  test_results <- perform_wilcoxon_test(var, "type")
  letter <- letters[i]
  p <- plot_wilcoxon_results(data, var, test_results$p_value, letter)
  plot_list[[var]] <- p

  # Add results to data frame
  wilcoxon_results <- rbind(wilcoxon_results, data.frame(
    Variable = var,
    W_Statistic = test_results$statistic,
    P_Value = test_results$p_value,
    Group1_Median = test_results$group1_median,
    Group1_IQR = test_results$group1_iqr,
    Group2_Median = test_results$group2_median,
    Group2_IQR = test_results$group2_iqr,
    Group1_Name = test_results$group1_name,
    Group2_Name = test_results$group2_name,
    stringsAsFactors = FALSE
  ))
}

# Arrange plots on one page
combined_plot <- do.call(grid.arrange, c(plot_list, ncol = 4)) # ncol can be adjusted as desired

# Save combined plot
ggsave("Wilcoxon_Test_Results_Plot.png", combined_plot, width = 16, height = 12, dpi = 300)
ggsave("Wilcoxon_Test_Results_Plot.pdf", combined_plot, width = 16, height = 12)

# Save Wilcoxon test results to CSV file
write.csv(wilcoxon_results, "Wilcoxon_Test_Results_Statistics.csv", row.names = FALSE)

# Optional: Add FDR correction
wilcoxon_results$FDR_Adjusted_P <- p.adjust(wilcoxon_results$P_Value, method = "fdr")
write.csv(wilcoxon_results,   "Wilcoxon_Test_Results_Statistics_with_FDR.csv",   row.names   =
FALSE)

# Output result summary to console
cat("Wilcoxon rank sum test result summary:\n")
```

```
cat("============================\n")
print(wilcoxon_results[, c("Variable", "P_Value", "FDR_Adjusted_P")])

cat("\nPlots saved as: Wilcoxon_Test_Results_Plot.png and Wilcoxon_Test_Results_Plot.pdf\n")
cat("Statistical results saved as: Wilcoxon_Test_Results_Statistics.csv and Wilcoxon_Test_Results_Statistics_with_FDR.csv\n")


#### Wilcoxon rank sum test plotting code including healthy reference intervals ###
setwd("D:/ADV_healthy")

# Read data
packageVersion("ggplot2")

library(ggplot2)
library(dplyr)
library(grid)
library(gridExtra)

# Read data
data <- read.csv("D:/ADV_healthy/Healthy_Wilcoxon rank sum test data.csv")

# Replace special characters in column names
names(data) <- gsub("—", "-", names(data))

# Ensure 'type' column in data is a factor with correct order
data$type <- factor(data$type, levels = c("NSAP", "SAP", "Healthy"))

# Define reference range data
reference_ranges <- data.frame(
  indicator = c("PCT", "IFN", "IL.2", "IL.4", "IL.6", "IL.10", "TNF"),
  lower = c(0.07, 1.1, 1.1, 0.1, 1.7, 2.6, 0.1),
  upper = c(0.35, 17.3, 9.8, 4, 16.6, 4.9, 5.2)
)

# Define a function to perform Wilcoxon rank sum test (comparing only SAP and NSAP)
perform_wilcoxon_test <- function(var, group) {
  # Use only patient data for comparison, exclude healthy group
  patient_data <- data[data$type %in% c("NSAP", "SAP"), ]
  test_result <- wilcox.test(patient_data[[var]] ~ patient_data[[group]])
  return(test_result$p.value)
}

# Define plotting function - modified to add sample size, implement italic p, and superscript
```

```
in Y-axis units
plot_wilcoxon_results <- function(data, var, p_value, letter, reference_ranges) {
  # Create dynamic Y-axis title - use expression from second code to implement superscript
  y_label <- switch(var,
                    "PLT" = expression(PLT ~ (10^9/L)),
                    "CRP" = "CRP (mg/L)",
                    "PA" = "PA (mg/L)",
                    "IFN" = "IFN-γ (pg/mL)",
                    "PLR" = "PLR",
                    "CPAR" = "CPAR",
                    "PCT" = "PCT (ng/mL)",
                    "PNR" = "PNR",
                    "NLR" = "NLR",
                    "LYM" = expression(LYM ~ (10^9/L)),
                    "NEV" = expression(NEV ~ (10^9/L)),
                    "IL.2" = "IL-2 (pg/mL)",
                    "IL.4" = "IL-4 (pg/mL)",
                    "IL.6" = "IL-6 (pg/mL)",
                    "IL.10" = "IL-10 (pg/mL)",
                    "TNF" = "TNF (pg/mL)",
                    var)

  # Create color mapping: patient groups in blue shades, healthy group in green
  fill_colors <- c("NSAP" = "#FFFF00", "SAP" = "#FF0000", "Healthy" = "#008000")

  # Check if this indicator has actual healthy population data
  healthy_subset <- subset(data, type == "Healthy")
  has_healthy_data <- any(!is.na(healthy_subset[[var]]))

  # Check if this indicator has a reference range
  has_reference_range <- any(reference_ranges$indicator == var)

  # Calculate sample size for each group (number of non-missing values) - keep sample size
calculation from first code
  sample_sizes <- data %>%
    group_by(type) %>%
    summarise(
      n = sum(!is.na(.data[[var]])),
      .groups = 'drop'
    )

  # Create labels with sample sizes for each group
  x_labels <- c()
  for(level in levels(data$type)) {
```

```
    n_val <- sample_sizes$n[sample_sizes$type == level]
    if(length(n_val) > 0) {
      x_labels[level] <- paste0(level, "\n(n=", n_val, ")")
    } else {
      x_labels[level] <- level
    }
  }

  # Create base plot - only plot boxplots for patient groups
  p <- ggplot(data, aes(x = type, y = .data[[var]], fill = type)) +
    # Draw boxplots for patient groups
    geom_boxplot(data = subset(data, type %in% c("NSAP", "SAP"))) +
    scale_fill_manual(values = fill_colors) +
    scale_x_discrete(labels = x_labels) +   # Use x-axis labels with sample sizes
    # Use bquote from second code to implement italic p-value
    labs(title = bquote(italic(p) ~ "=" ~ .(sprintf("%.4f", p_value))),
         x = NULL,
         y = y_label) +
    theme_minimal() +
    theme(
      plot.title = element_text(size = 14, hjust = 0.5),
      axis.title.x = element_text(size = 12),
      axis.title.y = element_text(size = 12),
      axis.text.x = element_text(size = 10, lineheight = 0.8),   # Adjust x-axis text size and
line height
      axis.text.y = element_text(size = 10),
      legend.position = "none",
      panel.grid.major = element_blank(),
      panel.grid.minor = element_blank(),
      axis.line = element_line(color = "black"),
      axis.ticks.y = element_line(color = "black", size = 0.5),
      plot.margin = margin(5, 5, 5, 5, "mm")
    ) +
    # Place letter identifier on the left side of Y-axis
    annotate("text", x = 0, y = Inf, label = letter,
             size = 5, fontface = "bold", color = "black",
             hjust = 1, vjust = -0.5) +
    coord_cartesian(clip = 'off')

  # Add healthy population data based on availability
  if (has_healthy_data) {
    # Has actual healthy population data - use boxplot
    p <- p + geom_boxplot(data = subset(data, type == "Healthy"))
  } else if (has_reference_range) {
```

```r
    # Only has reference range - use rectangle to represent reference range
    ref_row <- reference_ranges[reference_ranges$indicator == var, ]
    if (nrow(ref_row) > 0) {
      # Calculate rectangle position (x-axis position corresponds to Healthy group)
      x_min <- 2.6    # Starting position of Healthy group on x-axis
      x_max <- 3.4    # Ending position of Healthy group on x-axis

      p <- p +
        # Draw reference range rectangle
        geom_rect(aes(xmin = x_min, xmax = x_max,
                      ymin = ref_row$lower, ymax = ref_row$upper),
                  fill = "#A1D99B", alpha = 0.7, color = "darkgreen", size = 0.5,
                  inherit.aes = FALSE) +
        # Add reference range label
        annotate("text", x = 3, y = (ref_row$lower + ref_row$upper)/2,
                 label = "Ref", size = 3, color = "darkgreen")
    }
  }

  return(p)
}

# Iterate over all variables and perform Wilcoxon rank sum test and plotting
plot_list <- list()
letters <- LETTERS[1:16]

# Ensure all data column names exclude 'type'
variable_names <- names(data)[!names(data) %in% "type"]

for (i in seq_along(variable_names)) {
  var <- variable_names[i]
  p_value <- perform_wilcoxon_test(var, "type")
  letter <- letters[i]
  p <- plot_wilcoxon_results(data, var, p_value, letter, reference_ranges)
  plot_list[[var]] <- p
}

# Arrange plots on one page
final_plot <- do.call(grid.arrange, c(plot_list, ncol = 4))

# Use ggsave to save high-resolution image
ggsave(filename = "perfect_Figure2_With_Sample_Sizes_Improved.tiff",
       plot = final_plot,
       dpi = 600,
```

```r
            width = 16,    # Increase width to accommodate sample size labels
            height = 12,
            units = "in",
            compression = "lzw")

# Also save as PDF format
ggsave(filename = "Figure2_With_Sample_Sizes_Improved.pdf",
            plot = final_plot,
            width = 16,
            height = 12)

cat("Improved Figure 2 successfully generated and saved, includes sample size information,
italic p-value and correct superscript display\n")
cat("Files saved as: Figure2_With_Sample_Sizes_Improved.tiff and
Figure2_With_Sample_Sizes_Improved.pdf\n")

####### Plot ROC curves for 6 indicators with P<0.05 and calculate AUC with 95% confidence
intervals #####

setwd("D:/ADV_healthy")

library(pROC)

# Read CSV file
data <- read.csv("D:\\ADV\\ROCtotal.csv")

# Convert disease type to binary factor
data$type <- factor(data$type, levels = c("NSAP", "SAP"))

# Calculate case numbers for each indicator separately (in original data)
n_cases <- c(
  CRP = sum(!is.na(data$CRP) & !is.na(data$type)),
  PA = sum(!is.na(data$PA) & !is.na(data$type)),
  IFN = sum(!is.na(data$IFN) & !is.na(data$type)),
  PLT = sum(!is.na(data$PLT) & !is.na(data$type)),
  PLR = sum(!is.na(data$PLR) & !is.na(data$type)),
  CPAR = sum(!is.na(data$CPAR) & !is.na(data$type))
)

# Process ROC curve calculation for each indicator separately
# For each indicator, only remove missing values for that indicator and type
roc1 <- roc(data$type[!is.na(data$CRP) & !is.na(data$type)],
                    data$CRP[!is.na(data$CRP) & !is.na(data$type)])
roc2 <- roc(data$type[!is.na(data$PA) & !is.na(data$type)],
```

```
                  data$PA[!is.na(data$PA) & !is.na(data$type)])
roc3 <- roc(data$type[!is.na(data$IFN) & !is.na(data$type)],
                  data$IFN[!is.na(data$IFN) & !is.na(data$type)])
roc4 <- roc(data$type[!is.na(data$PLT) & !is.na(data$type)],
                  data$PLT[!is.na(data$PLT) & !is.na(data$type)])
roc5 <- roc(data$type[!is.na(data$PLR) & !is.na(data$type)],
                  data$PLR[!is.na(data$PLR) & !is.na(data$type)])
roc6 <- roc(data$type[!is.na(data$CPAR) & !is.na(data$type)],
                  data$CPAR[!is.na(data$CPAR) & !is.na(data$type)])

# Calculate 95% confidence intervals for AUC
auc1_ci <- ci.auc(roc1)
auc2_ci <- ci.auc(roc2)
auc3_ci <- ci.auc(roc3)
auc4_ci <- ci.auc(roc4)
auc5_ci <- ci.auc(roc5)
auc6_ci <- ci.auc(roc6)

# Set high-resolution output
png("ROC_Curve_HighRes.png", width = 1200, height = 1000, res = 150)

# Reset graphics parameters
dev.off()
graphics.off()
par(pty="s", mar = c(5, 5, 4, 12))

# Set plotting area to square and draw ROC curves
plot(roc1, col="red", lwd=2, main="ROC Curves for Indicators with P<0.05",
       cex.axis=1.0, cex.lab=1.2)
lines(roc2, col="blue", lwd=2)
lines(roc3, col="orange", lwd=2)
lines(roc4, col="purple", lwd=2)
lines(roc5, col="#66CC00", lwd=2)
lines(roc6, col="#FF00FF", lwd=2)

# Add diagonal reference line
abline(a=0, b=1, col="gray", lty=2)

# Correct AUC value calculation
auc1 <- round(roc1$auc, 3)
auc2 <- round(roc2$auc, 3)
auc3 <- round(roc3$auc, 3)
auc4 <- round(roc4$auc, 3)
auc5 <- round(roc5$auc, 3)
```

```
auc6 <- round(roc6$auc, 3)

# Create legend text including AUC, confidence intervals and case numbers - using en dash
legend_text <- c(
  paste0("CRP: ", auc1, " (", round(auc1_ci[1], 3), "\u2013", round(auc1_ci[3], 3), ")\nn=", n_cases["CRP"]),
  paste0("PA: ", auc2, " (", round(auc2_ci[1], 3), "\u2013", round(auc2_ci[3], 3), ")\nn=", n_cases["PA"]),
  paste0("IFN-\u03b3: ", auc3, " (", round(auc3_ci[1], 3), "\u2013", round(auc3_ci[3], 3), ")\nn=", n_cases["IFN"]),
  paste0("PLT: ", auc4, " (", round(auc4_ci[1], 3), "\u2013", round(auc4_ci[3], 3), ")\nn=", n_cases["PLT"]),
  paste0("PLR: ", auc5, " (", round(auc5_ci[1], 3), "\u2013", round(auc5_ci[3], 3), ")\nn=", n_cases["PLR"]),
  paste0("CPAR: ", auc6, " (", round(auc6_ci[1], 3), "\u2013", round(auc6_ci[3], 3), ")\nn=", n_cases["CPAR"])
)

# Use precise coordinates to position legend
legend(x = 0.45, y = 0.35,
       legend = legend_text,
       col = c("red", "blue", "orange", "purple", "#66CC00", "#FF00FF"),
       lwd = 2,
       cex = 0.6,
       bg = NA,
       box.lwd = 0.5,
       x.intersp = 0.3,
       y.intersp = 0.4,
       text.width = 0.25,
       xpd = TRUE)

# Output AUC, confidence intervals and case numbers to console - also using en dash
cat("=== AUC, 95% Confidence Intervals and Case Numbers ===\n")
cat("CRP: AUC =", auc1, "95% CI:", round(auc1_ci[1], 3), "\u2013", round(auc1_ci[3], 3), "n =", n_cases["CRP"], "\n")
cat("PA: AUC =", auc2, "95% CI:", round(auc2_ci[1], 3), "\u2013", round(auc2_ci[3], 3), "n =", n_cases["PA"], "\n")
cat("IFN: AUC =", auc3, "95% CI:", round(auc3_ci[1], 3), "\u2013", round(auc3_ci[3], 3), "n =", n_cases["IFN"], "\n")
cat("PLT: AUC =", auc4, "95% CI:", round(auc4_ci[1], 3), "\u2013", round(auc4_ci[3], 3), "n =", n_cases["PLT"], "\n")
cat("PLR: AUC =", auc5, "95% CI:", round(auc5_ci[1], 3), "\u2013", round(auc5_ci[3], 3), "n =", n_cases["PLR"], "\n")
cat("CPAR: AUC =", auc6, "95% CI:", round(auc6_ci[1], 3), "\u2013", round(auc6_ci[3], 3), "n =",
```

n_cases["CPAR"], "\n")
cat("Total valid cases:", sum(n_cases > 0), "\n")

random forest implementation

```
#### Code with confidence intervals added on 10.22 ###
setwd("D:\\ADV")
# Read CSV file
library(data.table)
data <- fread("D:/ADV/new_model.csv")

# Load required packages
library(data.table)
library(randomForest)
library(caret)
library(pROC)
library(ggplot2)
library(dplyr)
library(boot)   # Add boot package for bootstrap confidence intervals

# View data structure
str(data)

# Convert first column (classification labels) to factor
data[, type := as.factor(type)]

# Handle missing values - extend to all features
numeric_columns <- c("PLT", "CRP", "PA", "IFN", "CPAR", "PLR", "PCT", "PNR", "NLR",
                     "LYM", "NEV", "IL-2", "IL-4", "IL-6", "IL-10", "TNF")

for(col in numeric_columns) {
  if(col %in% names(data)) {
    data[[col]] <- ifelse(is.na(data[[col]]), mean(data[[col]], na.rm = TRUE), data[[col]])
  }
}

# Extract all candidate features and labels
all_features <- data[, .SD, .SDcols = numeric_columns]
labels <- data$type

# Check class distribution
cat("Class distribution:\n")
print(table(labels))
```

```r
cat("\n")

# ========== New: Biomarker statistical description ==========
cat("=== Biomarker Statistical Description ===\n")
biomarker_summary <- function(feature_data, feature_name) {
  data.frame(
    Feature = feature_name,
    Mean = round(mean(feature_data, na.rm = TRUE), 2),
    SD = round(sd(feature_data, na.rm = TRUE), 2),
    Median = round(median(feature_data, na.rm = TRUE), 2),
    IQR_25 = round(quantile(feature_data, 0.25, na.rm = TRUE), 2),
    IQR_75 = round(quantile(feature_data, 0.75, na.rm = TRUE), 2),
    N = sum(!is.na(feature_data))
  )
}

# Apply to each feature
stats_list <- lapply(numeric_columns, function(col) {
  biomarker_summary(data[[col]], col)
})
biomarker_stats <- do.call(rbind, stats_list)
print(biomarker_stats)
write.csv(biomarker_stats, file = "biomarker_descriptive_statistics.csv", row.names = FALSE)
cat("\n")

# Split training and test sets
set.seed(123)
train_index <- sample(seq_len(nrow(data)), size = 0.7 * nrow(data))

# Perform feature selection within training set only
train_data_for_selection <- all_features[train_index, ]
train_labels_for_selection <- labels[train_index]

# Perform univariate significance testing (only on training set!)
p_values <- sapply(train_data_for_selection, function(feature) {
  if(is.numeric(feature)) {
    wilcox.test(feature ~ train_labels_for_selection)$p.value
  }
})

# Apply multiple hypothesis testing correction (FDR)
adjusted_p_values <- p.adjust(p_values, method = "fdr")
significant_features <- names(adjusted_p_values[adjusted_p_values < 0.05])
```

```
cat("Features significant on training set (after FDR correction):\n")
feature_selection_info <- data.frame(
  Feature = names(p_values),
  P_Value = p_values,
  Adjusted_P_Value = adjusted_p_values,
  Significant = adjusted_p_values < 0.05
)
print(feature_selection_info)

# If no features are significant, select top 3 features with smallest adjusted p-values
if(length(significant_features) == 0) {
  warning("No features found significant on training set after FDR correction, using top 3
features with smallest adjusted p-values")
  significant_features <- names(sort(adjusted_p_values)[1:min(3, length(adjusted_p_values))])
}

# Use selected features
selected_features <- all_features[, ..significant_features]
train_features <- selected_features[train_index, ]
test_features <- selected_features[-train_index, ]
train_labels <- labels[train_index]
test_labels <- labels[-train_index]

# Set up repeated cross-validation and handle class imbalance
ctrl <- trainControl(
  method = "repeatedcv",       # Repeated cross-validation
  number = 5,                   # 5-fold
  repeats = 10,                # Repeat 10 times
  classProbs = TRUE,
  summaryFunction = twoClassSummary,
  sampling = "up",             # Up-sampling to handle class imbalance
  savePredictions = TRUE,
  verboseIter = FALSE
)

# More detailed parameter tuning grid
tuneGrid <- expand.grid(
  mtry = c(2, floor(sqrt(ncol(train_features))), floor(ncol(train_features)/2), ncol(train_features))
)

# Random forest model training (includes repeated cross-validation and up-sampling)
set.seed(123)
rf_model <- train(
  x = train_features,
```

```
  y = train_labels,
  method = "rf",
  trControl = ctrl,
  metric = "ROC",
  ntree = 500,                    # Increase number of trees
  tuneGrid = tuneGrid,
  importance = TRUE
)

print(rf_model)

# View cross-validation results
cat("Cross-validation results:\n")
print(rf_model$results)

# View best parameters
cat("Best parameters:\n")
print(rf_model$bestTune)

# ========== New: Calculate cross-validation AUC confidence interval ==========
cv_auc_ci <- quantile(rf_model$resample$ROC, c(0.025, 0.975))
cat("Cross-validation AUC 95% confidence interval:", round(cv_auc_ci[1], 3), "-", round(cv_auc_ci[2], 3), "\n")

# Evaluate model on test set
rf_predictions <- predict(rf_model, newdata = test_features)
rf_probs <- predict(rf_model, newdata = test_features, type = "prob")

# Ensure factor levels are consistent
rf_predictions <- factor(rf_predictions, levels = levels(test_labels))

# Calculate confusion matrix
rf_conf_matrix <- confusionMatrix(rf_predictions, test_labels)
print(rf_conf_matrix)

# Calculate balanced accuracy
balanced_accuracy <- (rf_conf_matrix$byClass['Sensitivity'] + rf_conf_matrix$byClass['Specificity']) / 2

# Calculate MCC (Matthews Correlation Coefficient)
if(!require(mltools)) {
  install.packages("mltools")
  library(mltools)
}
```

```r
mcc_value <- mcc(rf_predictions, test_labels)

# Extract evaluation metrics
accuracy <- rf_conf_matrix$overall['Accuracy']
precision <- rf_conf_matrix$byClass['Pos Pred Value']
recall <- rf_conf_matrix$byClass['Sensitivity']
specificity <- rf_conf_matrix$byClass['Specificity']
f1 <- rf_conf_matrix$byClass['F1']
kappa <- rf_conf_matrix$overall['Kappa']

# Create detailed evaluation metrics table
evaluationMetrics <- data.frame(
  Metric = c("Accuracy", "Sensitivity", "Specificity", "Precision",
             "F1 Score", "Balanced Accuracy", "Kappa", "MCC", "AUC"),
  Value = c(accuracy, recall, specificity, precision,
            f1, balanced_accuracy, kappa, mcc_value, NA)
)

# Calculate ROC curve and AUC
roc_obj <- roc(test_labels, rf_probs[, 2])
auc_value <- auc(roc_obj)
evaluationMetrics$Value[evaluationMetrics$Metric == "AUC"] <- auc_value

# ========== New: Calculate test set AUC confidence interval ==========
roc_ci <- ci.auc(roc_obj)
cat("Test set AUC 95% confidence interval:", round(roc_ci[1], 3), "-", round(roc_ci[3], 3), "\n")

# Update evaluation metrics table to include confidence intervals
evaluationMetrics$CI_Lower <- NA
evaluationMetrics$CI_Upper <- NA
evaluationMetrics$CI_Lower[evaluationMetrics$Metric == "AUC"] <- roc_ci[1]
evaluationMetrics$CI_Upper[evaluationMetrics$Metric == "AUC"] <- roc_ci[3]

cat("Performance on independent test set:\n")
print(evaluationMetrics)

# ========== New: Calculate bootstrap confidence intervals for other metrics ==========
cat("\n=== Calculating bootstrap confidence intervals for other metrics ===\n")
calculate_bootstrap_ci <- function(data, indices, metric_func) {
  sample_preds <- data$predictions[indices]
  sample_labels <- data$labels[indices]
  return(metric_func(sample_preds, sample_labels))
}
```

```
# Prepare data
boot_data <- data.frame(predictions = rf_predictions, labels = test_labels)

# Bootstrap CI for accuracy
boot_accuracy <- boot(boot_data, statistic = calculate_bootstrap_ci, R = 1000,
                      metric_func = function(preds, labels) mean(preds == labels))
accuracy_ci <- boot.ci(boot_accuracy, type = "perc")
cat("Accuracy 95% CI:", round(accuracy_ci$percent[4], 3), "-", round(accuracy_ci$percent[5], 3),
"\n")

# Bootstrap CI for sensitivity
boot_sensitivity <- boot(boot_data, statistic = calculate_bootstrap_ci, R = 1000,
                         metric_func = function(preds, labels) {
                           conf_matrix <- table(preds, labels)
                           if(ncol(conf_matrix) == 2) {
                             return(conf_matrix[1,1] / (conf_matrix[1,1] +
conf_matrix[2,1]))
                           } else {
                             return(NA)
                           }
                         })
sensitivity_ci <- boot.ci(boot_sensitivity, type = "perc")
cat("Sensitivity 95% CI:", round(sensitivity_ci$percent[4], 3), "-", round(sensitivity_ci$percent[5],
3), "\n")

# Feature importance (based on final model)
final_rf <- rf_model$finalModel
importance_values <- importance(final_rf)
importance_df <- as.data.frame(importance_values)
importance_df$Feature <- rownames(importance_df)

# Plot feature importance
p_importance <- ggplot(importance_df, aes(x = reorder(Feature, MeanDecreaseGini), y =
MeanDecreaseGini)) +
  geom_bar(stat = "identity", fill = "#D2691E", width = 0.5) +
  geom_text(aes(label = round(MeanDecreaseGini, 2)),
            position = position_stack(vjust = 0.5),
            vjust = -0.5, size = 5) +
  coord_flip() +
  labs(title = "Feature Importance (After FDR Correction and Up-sampling)",
       x = "Feature", y = "Mean Decrease in Gini") +
  theme(
    plot.title = element_text(size = 16),
```

```
      axis.title.x = element_text(size = 14),
      axis.title.y = element_text(size = 14),
      axis.text.x = element_text(size = 12),
      axis.text.y = element_text(size = 12)
  )
print(p_importance)

# ========== Modified: Plot ROC curve with confidence intervals ==========
# Set graphics to square
par(pty = "s")

plot(roc_obj,
     main = paste("Random Forest ROC Curve\nAUC =",
                  round(auc_value, 3),
                  "95% CI:",
                  round(roc_ci[1], 3), "\u2013", round(roc_ci[3], 3)),
     col = "#006400", lwd = 3, cex.axis = 1.0, cex.lab = 1.5)   # Dark green
abline(a = 0, b = 1, col = "gray", lty = 2)

# Adjust legend position and size, using transparent background
legend(x = 0.393, y = 0.152,   # Use precise coordinates to position legend, bottom-right
       legend = c(paste("AUC =", round(auc_value, 3)),
                  paste("95% CI:", round(roc_ci[1], 3), "\u2013", round(roc_ci[3], 3)),
                  paste("n =", length(test_labels))),
       col = c("#006400", "black", "black"),   # Only first line uses dark green
       lty = c(1, 0, 0),                        # Only first line shows line
       lwd = c(2, 0, 0),                        # Only first line shows line width
       seg.len = 1,                             # Line segment length
       cex = 0.6,                               # Font size, consistent with before
       bg = NA,                                 # Transparent background
       box.lwd = 0.5,                           # Box line width, consistent with before
       x.intersp = 0.3,                         # Horizontal spacing, consistent with before
       y.intersp = 0.4,                         # Vertical spacing, consistent with before
       text.width = 0.25,                       # Text width, consistent with before
       xpd = TRUE)                              # Allow plotting outside figure

# Restore default graphics parameters
par(pty = "m")

# ####========== Plot performance metrics comparison chart
metrics_plot <- evaluationMetrics %>%
  filter(Metric %in% c("Accuracy", "Sensitivity", "Specificity", "F1 Score", "Balanced
Accuracy")) %>%
  ggplot(aes(x = Metric, y = Value, fill = Metric)) +
```

```r
  geom_bar(stat = "identity", alpha = 0.8) +
  geom_text(aes(label = round(Value, 3)), vjust = -0.5, size = 4) +
  ylim(0, 1) +
  labs(title = "Model Performance Metrics", y = "Score") +
  theme_minimal() +
  theme(axis.text.x = element_text(angle = 45, hjust = 1))
print(metrics_plot)

# Save important results
write.csv(evaluationMetrics, file = "evaluation_metrics_comprehensive.csv", row.names =
FALSE)
write.csv(importance_df, file = "feature_importance_comprehensive.csv", row.names = FALSE)
write.csv(feature_selection_info, file = "feature_selection_process_fdr.csv", row.names = FALSE)

# Save cross-validation results
cv_results <- rf_model$resample
write.csv(cv_results, file = "cross_validation_results.csv", row.names = FALSE)

# ========== New: Save confidence interval results ==========
ci_results <- data.frame(
  Metric = c("Test_Set_AUC", "CV_AUC", "Accuracy", "Sensitivity"),
  Estimate = c(auc_value, mean(rf_model$resample$ROC), accuracy, recall),
  CI_Lower = c(roc_ci[1], cv_auc_ci[1], accuracy_ci$percent[4], sensitivity_ci$percent[4]),
  CI_Upper = c(roc_ci[3], cv_auc_ci[2], accuracy_ci$percent[5], sensitivity_ci$percent[5])
)
write.csv(ci_results, file = "confidence_intervals.csv", row.names = FALSE)

# Output detailed summary
cat("\n=== Comprehensive Analysis Summary ===\n")
cat("Initial number of features:", ncol(all_features), "\n")
cat("Number of significant features after FDR correction:", length(significant_features), "\n")
cat("Features used:", paste(significant_features, collapse = ", "), "\n")
cat("Class imbalance handling: Up-sampling\n")
cat("Cross-validation: 10 repeats of 5-fold cross-validation\n")
cat("Multiple testing correction: FDR method\n")
cat("Test set sample size:", length(test_labels), "\n")
cat("Training set sample size:", length(train_labels), "\n")
cat("Class distribution - Training set: \n")
print(table(train_labels))
cat("Class distribution - Test set: \n")
print(table(test_labels))

# ========== New: Output confidence interval summary ==========
cat("\n=== Confidence Interval Summary ===\n")
```

```
cat("Test set AUC:", round(auc_value, 3), "95% CI:", round(roc_ci[1], 3), "-", round(roc_ci[3], 3), "\n")
cat("Cross-validation AUC:", round(mean(rf_model$resample$ROC), 3), "95% CI:", round(cv_auc_ci[1], 3), "-", round(cv_auc_ci[2], 3), "\n")
cat("Accuracy:", round(accuracy, 3), "95% CI:", round(accuracy_ci$percent[4], 3), "-", round(accuracy_ci$percent[5], 3), "\n")
cat("Sensitivity:", round(recall, 3), "95% CI:", round(sensitivity_ci$percent[4], 3), "-", round(sensitivity_ci$percent[5], 3), "\n")

cat("\nMain improvements:\n")
cat("1. Applied FDR multiple testing correction\n")
cat("2. Used up-sampling to handle class imbalance\n")
cat("3. Employed repeated cross-validation (5-fold 10 times)\n")
cat("4. Added more comprehensive evaluation metrics (Balanced Accuracy, MCC)\n")
cat("5. More detailed parameter tuning grid\n")
cat("6. Added 95% confidence intervals for all AUC values\n")
cat("7. Added detailed biomarker statistical description\n")
cat("8. Added bootstrap confidence intervals for other key metrics\n")
```

```
plot(roc_obj,
     main = paste("Random Forest ROC Curve\nAUC =",
                  round(auc_value, 3),
                  "95% CI:",
                  round(roc_ci[1], 3), "\u2013", round(roc_ci[3], 3)),
     col = "#006400", lwd = 3, cex.axis = 1.0, cex.lab = 1.5)  # Dark green
abline(a = 0, b = 1, col = "gray", lty = 2)

# Adjust legend position and size to avoid covering curve
legend("bottomright",
       legend = c(paste("AUC =", round(auc_value, 3)),
                  paste("95% CI:", round(roc_ci[1], 3), "\u2013", round(roc_ci[3], 3)),
                  paste("n =", length(test_labels))),
       col = c("#006400", "black", "black"),  # Only first line uses dark green
       lty = c(1, 0, 0),                      # Only first line shows line
       lwd = c(2, 0, 0),                      # Only first line shows line width
       seg.len = 1,                           # Line segment length
       cex = 0.8,                             # Reduce font size
       bty = "n",                             # Remove legend box
       inset = c(0.02, 0.02),                 # Move slightly inward to avoid edges
       x.intersp = 0.5,                       # Reduce horizontal spacing
       y.intersp = 0.8)                       # Reduce vertical spacing
```

```r
# ========== Modified: Plot ROC curve with confidence intervals ==========
# ========== Modified: Plot ROC curve with confidence intervals ==========
# Set graphics to square
par(pty = "s")

plot(roc_obj,
     main = paste("Random Forest ROC Curve\nAUC =",
                  round(auc_value, 3),
                  "95% CI:",
                  round(roc_ci[1], 3), "\u2013", round(roc_ci[3], 3)),
     col = "#006400", lwd = 3, cex.axis = 1.0, cex.lab = 1.5)   # Dark green
abline(a = 0, b = 1, col = "gray", lty = 2)

# Adjust legend position and size, using transparent background
legend(x = 0.393, y = 0.152,   # Use precise coordinates to position legend, bottom-right
       legend = c(paste("AUC =", round(auc_value, 3)),
                  paste("95% CI:", round(roc_ci[1], 3), "\u2013", round(roc_ci[3], 3)),
                  paste("n =", length(test_labels))),
       col = c("#006400", "black", "black"),   # Only first line uses dark green
       lty = c(1, 0, 0),                        # Only first line shows line
       lwd = c(2, 0, 0),                        # Only first line shows line width
       seg.len = 1,                             # Line segment length
       cex = 0.6,                               # Font size, consistent with before
       bg = NA,                                 # Transparent background
       box.lwd = 0.5,                           # Box line width, consistent with before
       x.intersp = 0.3,                         # Horizontal spacing, consistent with before
       y.intersp = 0.4,                         # Vertical spacing, consistent with before
       text.width = 0.25,                       # Text width, consistent with before
       xpd = TRUE)                              # Allow plotting outside figure

# Restore default graphics parameters
par(pty = "m")
```
